# Clinical Perspective on Primary Angiitis of the Central Nervous System in Childhood (cPACNS)

**DOI:** 10.3389/fped.2020.00281

**Published:** 2020-07-03

**Authors:** Martin Smitka, Normi Bruck, Kay Engellandt, Gabriele Hahn, Ralf Knoefler, Maja von der Hagen

**Affiliations:** ^1^Abteilung Neuropädiatrie, Medizinische Fakultät Carl Gustav Carus, Technische Universität Dresden, Dresden, Germany; ^2^Klinik für Kinder und Jugendmedizin, Medizinische Fakultät Carl Gustav Carus, Technische Universität Dresden, Dresden, Germany; ^3^Department of Neuroradiology, University Hospital Carl Gustav Carus, Technische Universität Dresden, Dresden, Germany; ^4^Bereich Kinderradiologie, Medizinische Fakultät Carl Gustav Carus, Institut und Poliklinik für Radiologische Diagnostik, Technische Universität Dresden, Dresden, Germany

**Keywords:** inflammatory brain disease, cerebral vasculitis in children, pediatric acute ischemic stroke, primary angiitis of the CNS in children cPACNS, immunomodulative therapy, cerebral arteriopathies, vascular imaging

## Abstract

Non-arteriosclerotic arteriopathies have emerged as important underlying pathomechanism in pediatric arterial ischemic stroke (AIS). The pathogenesis and classification of cerebral arteriopathies in childhood are heterogeneous. Different classifications base on (i) the anatomic site; (ii) the distribution and size of the affected vessel; (iii) the time course, for example, transient vs. progressive, monophasic vs. recurrent; (iv) the putative pathogenesis; (v) the magnetic resonance imaging morphology of the vasculopathies. Inflammation affecting the cerebral vessels is increasingly recognized as common cause of pediatric AIS. Primary cerebral vasculitis or primary angiitis of the central nervous system (CNS) in childhood (cPACNS) is an important differential diagnosis in pediatric AIS. Primary angiitis of the CNS is a rare disorder, and the pathogenesis is poorly understood so far. The current classification of cPACNS is based on the affected cerebral vessel size, the disease course, and angiographic pattern. Two large subtypes are currently recognized comprising large- and medium-sized vessel CNS vasculitis referred to as angiography-positive cPACNS and angiography-negative small vessel cPACNS. As the clinical manifestations of cPACNS are rather diverse, precise diagnosis can be challenging for the treating pediatrician because of the lack of vital laboratory tests or imaging features. Initial misdiagnosis is common because of overlapping phenotypes and pediatric AIS mimics. As untreated cPACNS is associated with a high morbidity and mortality, timely diagnosis, and induction of immunomodulatory and symptomatic therapy are essential. Survival and neurological outcome depend on early diagnosis and prompt therapy. Primary angiitis of the central nervous system in childhood differs in several aspects from primary cerebral angiitis in adults. The aim of this article is to give a brief comprehensive summary on pediatric primary cerebral vasculitis focusing on the clinical perspective regarding the classification, the putative pathogenesis, the disease course, the diagnostic tools, and emerging treatment options. A modified terminology for clinical practice is discussed.

## Background

Arterial ischemic stroke (AIS) is one of the most frequent causes of mortality and morbidity in adults and within the 10 leading causes of death in childhood. The estimated incidence of pediatric AIS is variable and ranges from 1.0 to 7.9 per 100,000 children beyond the neonatal period (1–5). The pathogenesis of pediatric AIS differs from those in adults, and the underlying mechanisms are poorly understood. Within the last two decades, the non-atherosclerotic arteriopathies have been increasingly recognized as the most prevalent etiology of pediatric AIS. Arteriopathy is an umbrella term covering a diverse group of conditions, including inflammatory angiitis, arterial dissection, moyamoya syndrome, and so on. Inflammation affecting the cerebral vessels is increasingly recognized as the most common cause of pediatric AIS. This review focuses on primary vasculitis or angiitis of the central nervous system (PACNS) in childhood (cPACNS). In adults, the prevalence of definite angiitis of the CNS causing an isolated presentation with ischemic stroke is estimated below 0.5% (6). The knowledge gap and heterogeneous nature of pediatric cerebral vasculopathy in conjunction with the low prevalence of stroke in childhood hamper the development of consensus-based international management guidelines, the identification of predictive prognostic markers, therapeutic approaches, and concepts for neurorehabilitation in pediatric AIS taking into account the developmental plasticity.

Based on predominant inflammatory mechanisms in angiitis of the central nervous system (CNS), the evaluation of immunomodulatory or immunosuppressive treatment comes into focus. As most case series suggest a high degree of good clinical outcome and absent recurrence in cPACNS when the patients are treated promptly with steroids and immunosuppressive therapy, a timely diagnosis is absolutely essential. The aim of this article is to give a brief comprehensive summary on pediatric primary cerebral vasculitis focusing on the clinical perspective regarding the classification, pathogenesis, clinical course, diagnostic pathways, and emerging treatment options.

### Classification of Pediatric Cerebral Vasculopathy and cPACNS

So far, there are limited data on the reliability of subtype classification in childhood AIS and cerebral vasculopathy. Nomenclature and definitions vary in between different clinical specializations, and similar magnetic resonance imaging (MRI) pattern of cerebral vasculopathies has been labeled differently. Different classifications base on the (i) anatomic site; (ii) the distribution; (iii) the time course, for example, transient vs. progressive, monophasic vs. recurrent; (iv) the putative pathogenesis; and (v) the MRI morphology of the vasculopathies. The International Pediatric Stroke Study created a consensus-based classification system, the CASCADE (Childhood AIS Standardized Classification and Diagnostic Evaluation) criteria (7). The primary CASCADE classification contains seven subtypes and is based on the anatomic site of disease including the heart, the great vessels of the neck, or the intracranial vessels. Secondary subtypes include additional information such as genetic causes of arteriopathy, hemoglobinopathy, or infections. The basic seven subtypes criteria comprise (i) the small vessel arteriopathy, (ii) the focal cerebral arteriopathy (FCA), (iii) the bilateral cerebral arteriopathy of childhood, (iv) the aortic/arteriopathy, (v) cardioembolic, (vi) other, and (vii) multifactorial (7). The CASCADE criteria have moderate reliability when used by trained and experienced raters, which suggests that it can be used for classification in multicenter pediatric stroke studies (8).

The most common intracranial arteriopathy in childhood presents as unilateral, focal stenosis in a focal cerebral arteriopathy (FCA) on cranial MRI (9, 10). Referring to the putative transient nature of the stenosis this specific monophasic, non-progressive arteriopathy is labeled transient cerebral arteriopathy (TCA) by the predominant neuropediatric specialists on childhood AIS (9–12). Focal cerebral arteriopathy and TCA is a descriptive diagnosis and does not specify the underlying pathophysiology. The VIPS (Vascular Effects of infection in Pediatric Stroke) study group emphasized the potential role of infection in TCA (13); furthermore, intracranial arterial dissection has been noted in TCA autopsy results (14). This emphasizes the assumption of a heterogeneous nature underlying pathophysiology in TCA.

Based on the presumed inflammatory pathogenesis, the predominantly rheumatologist group discusses primary and secondary angiitis of the CNS as putatively important cause of FCA (15–17). Primary angiitis of the central nervous system, also known as primary cerebral vasculitis, is a non-infectious, inflammatory disease that occurs in the CNS, involving the small and medium and pial vascular vessels of the brain.

**Figure 1:**
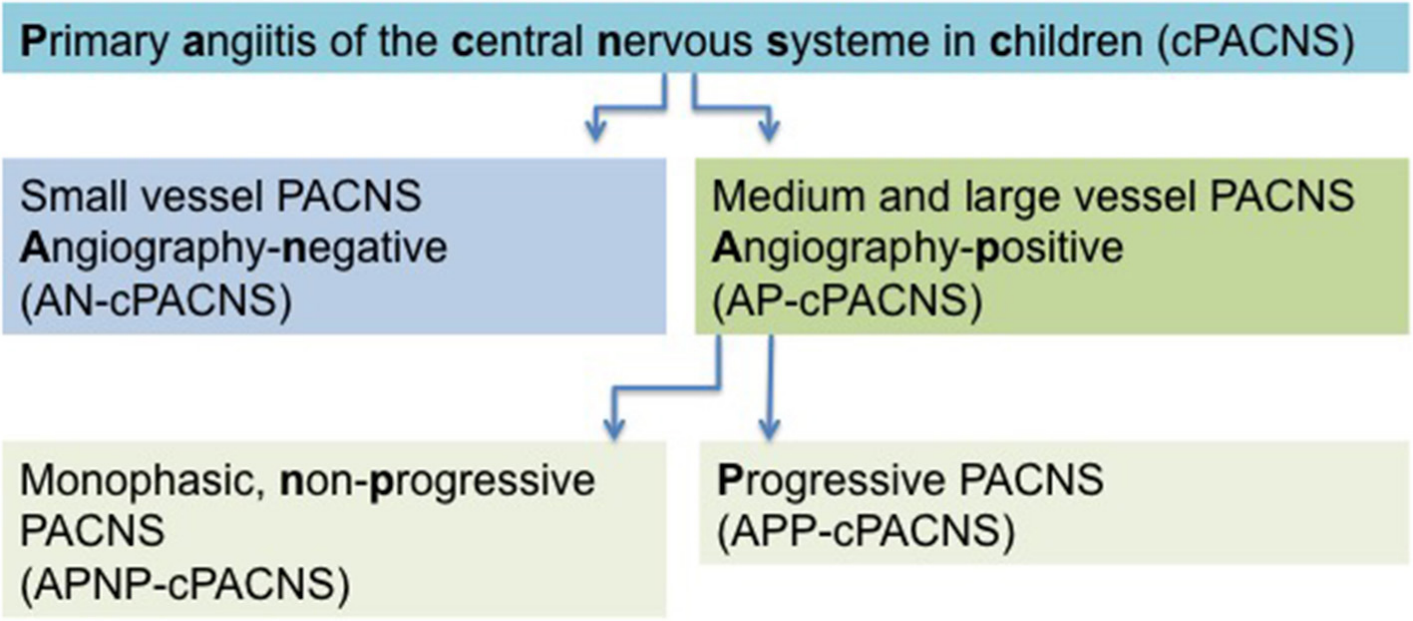
Classification scheme in cPACNS.

Analogous to the CASCADE criteria, the current classification of cPACNS is based on the affected cerebral vessel size and disease course. Three subtypes are recognized (Figure 1). Depending on the size of the affected vessel (large- and medium-sized vessel), CNS vasculitis is referred to as angiography-positive (AP-cPACNS) and angiography-negative small vessel PACNS (AN-cPACNS). Angiography-positive medium and large vessel cPACNS is further divided, non-progressive forms (APNP-cPACNS) and progressive forms (APP-cPACNS) (10, 15, 18, 19). The monophasic, non-progressive form is believed to account for the majority of patients with TCA. The three subtypes are associated with distinct presenting symptoms, pathogenesis, disease course, and treatment outcome. Demographic characteristics, clinical presentation, and brain histopathology of PACNS differ between adults and children (20). According to Calabrese and Mallek (21), the diagnosis of PACNS in adults requires three criteria: (i) an acquired and otherwise unexplained neurological deficit, (ii) classic angiographic or histopathological features of angiitis within the CNS, and (iii) the absence of another systemic disorder to explain these features. For pediatric patients (cPACNS), the criteria have been expanded to include acquired neurologic or psychiatric deficits (21, 22). In adults, Birnbaum and Hellmann (23) proposed to integrate the levels of certainty in the diagnosis of PACNS and suggest to apply (1) definite diagnosis of PACNS if there is confirmation of vasculitis by the analysis of tissue biopsy specimen, (2) probable diagnosis of PACNS in the absence of tissue if there are high-probability findings on an angiogram with abnormal findings on MRI and cerebrospinal fluid (CSF) profile consistent with PACNS.

Cerebral vasculitis as a consequence of an underlying systemic illness is classified as secondary CNS angiitis. Secondary CNS vasculitis may occur in the context of a systemic vasculitis, metabolic diseases, or as infection angiopathies. In childhood, an important differential diagnosis is post-varicella angiopathy (9, 12).

### Epidemiology

Estimates of the incidence of pediatric cerebral vasculitis are variable and highly dependent on the search strategy employed, as well as the study population. Furthermore, the lack of substantial agreement on the definition and labeling of pediatric cerebral vasculopathy explains the discrepancy between different epidemiological studies.

In adults, cerebral angiitis is a rare disorder. The annual incidence of PACNS has been estimated from 2: million to 4:1 million to fewer than 1:2 million in adults (24, 25). In adults, Kempster et al. (6) found a low prevalence of vasculitic stroke with 0.13%, and the prevalence of definite angiitis of the CNS causing an isolated presentation with ischemic stroke was 0.02%. In contrast to adulthood pediatric AIS itself is a rare disorder with an estimated incidence from 1.0 to 7.9 per 100,000 children beyond the neonatal period (1–5). Fullerton et al. (26) identified an underlying cPACNS in 24% of the cases with pediatric AIS, although the true prevalence in the cohort (*n* = 97 children with AIS) might be underestimated as 50% of the patients did not have vascular imaging. No incidence rates on pediatric PACNS are available.

## Pathogenesis

Childhood PACNS is an inflammatory brain diseases and is characterized by inflammation of cerebral blood vessels, classified by size (small, medium, large). The biological understanding of the underlying mechanism is still limited. There are numerous indicators for the inflammatory nature of this condition as histopathological evidence, MRI data, or cytokine/chemokine analysis in CSF. Histopathological examination in small-vessel vasculitis, for example, often reveals a lymphocytic vasculitis with a predominant intramural and perivascular T-cell infiltrate of the small muscular arteries, arterioles, capillaries, and venules (27). Magnetic resonance imaging reveals enhancement of the vessel wall together with thickening of the wall and parenchymal lesions. Cytokine/chemokine analysis can provide further insights into pathophysiological processes. Dabas and Yadav (28) analyzed cytokine/chemokine profiles in five children with stroke due to FCA in comparison to two children with arterial stroke due to other causes, 43 children with encephalitis, and 20 children with non-inflammatory neurological disease. This study revealed that levels for interleukin 6 (IL-6), IL-8, CXCL1, and CXCL10 were significantly higher in the acute CSF of FCA. The authors concluded that the results support innate, T-cell, and granulocyte inflammatory mechanisms in children with FCA (28). As FCA shows a significant overlap with the non-progressive form of AP-cPACNS, one might argue that this is another sign for the inflammatory nature of APNP-cPACNS. Another group studied different matrix metalloproteinases (MMPs), tissue inhibitors of MMPs, endothelial factors, vascular cell adhesion proteins, and cytokines in 12 children with AIS in comparison to neonatal stroke and healthy age-matched controls. At the time of the acute event, children with AIS had significantly elevated levels of MMP9, TIMP4 (tissue inhibitor of metalloprotease 4), IL-6, IL-8, and C-reactive protein (CRP) (29). Under the assumption that the majority of children with AIS have an underlying cPACNS, this kind of data can help to tailor immunosuppressive protocols in PACNS by increasing knowledge about underlying mechanisms. Valuable information for further insights will provide animal models as introduced by Faustino et al. (30).

Primary angiitis of the CNS in children can be secondary to a multitude of different conditions, including infectious diseases (e.g., human immunodeficiency virus, *Mycobacterium tuberculosis, Streptococcus*), post-infectious conditions (post-varicella angiopathy), and autoimmune and chronic systemic diseases (e.g., systemic lupus erythematosus, Takayasu arteritis). An overview about pathophysiological mechanisms in secondary angiitis is found in the study of Gowdie et al. (31).

## Clinical Features

The clinical presentation of PACNS is very heterogeneous, ranging from psychiatric and behavioral problems, transient minimal focal deficits, to persistent neurological symptoms (Table 1). The symptoms differ depending on the size of the affected vessels (medium- to large- vs. small-vessel vasculitis), on the involved brain areas, dimension of the inflammatory process, and other individual factors. In all inflammatory diseases of the brain in childhood, subacute or chronic headache is a key symptom of the disease. In an adult cohort with PACNS, the three main presenting symptoms were headache (60%), cerebral ischemia (75%), and altered cognition in 50%. Intracranial hemorrhage is infrequent (32, 33). From a clinical perspective, a definite differentiation between progressive and non-progressive AP-cPACNS is not entirely possible at manifestation. In APNP-cPACNS, patients can develop profound and permanent neurological deficits. In 60%, patients with APP-cPACNS develop permanent neurological deficits. Therefore, prompt initiation of therapy is strongly recommended after diagnosis of PACNS.

**Table 1:** Clinical course in medium- to large-vessel (AP-cPACNS) and small-vessel cerebral vasculitis (AN-cPACNS) in childhood.

	**APP-cPACNS**	**APNP-cPACNS**	**AN-cPACNS**
Onset of symptoms manifestations	Subacute/acute	Acute	Slowly progressive
Clinical presentation	Acute and subacute focal and systemic neurologic deficits, headache, encephalopathy, seizures, confusion, lethargy, impaired memory, unilateral, or bilateral stroke	Unilateral arterial ischemic stroke, acute focal neurologic deficit, headache, encephalopathy	Acute presentation with seizures, diffuse neurological deficits, signs of meningoencephalitis, encephalopathy, subacute and chronic presentation: psychiatric symptoms, learning difficulties, emotional instability, behavioral changes, memory impairment, headache, fever, fatigue
Sex	Male > female	Male > female	Female >> male

### Outcome

Long-term outcome of cPACNS has been studied only in small case series. Elbers et al. (34) showed in a cohort of 27 children with cPACNS after a 12-month observation period that 40% demonstrated progression on vascular imaging without clinical or angiographic predictors. On the other hand, abnormal vascular imaging in the sense of a cerebral arteriopathy is a strong predictor for a stroke recurrence. Ganesan et al. (35) found a 5-year cumulative recurrence in 66% of the children who had a stenotic vascular abnormality. Long-term follow-up MRI (24–72 months after disease onset) revealed progressive paucity of the peripheral vessels in ~70% of the children with cPACNS, which could be secondary to the inflammation affecting the peripheral vasculature (36). Most case series suggest a high degree of good clinical outcome and absent recurrence in cPACNS when the patients are treated with steroids and immunosuppressive therapy (37–39). Only a minority of children appears to develop a functional deficit after completing immunosuppressive treatment (37, 40).

Albeit in medium- and large-vessel-size cPACNS, the functional outcome, neurological sequelae, and recurrence rate are strongly correlated with pediatric AIS outcome and risk factors. Infarction pattern, for example, involving multiple different brain structures, unilaterally or bilaterally, and the age at manifestation of AIS correlate with disease impact. Clinical symptoms such as seizures at presentation of AIS were associated with a poor outcome (41–43) Childhood stroke in the context of preexisting critical illness has been reported to be a marker of increased mortality risk (44).

## Diagnostic Assessment

In the absence of timely diagnosis and immunosuppressive therapy, progressive inflammation in cPACNS results in an important morbidity and mortality (45, 46). When brain biopsy is unavailable or negative, diagnosis of AN-cPACNS relies on the combination of clinical features, brain imaging, and cerebral angiography, together with the exclusion of other more common diseases affecting CNS vessels (Table 2).

**Table 2:** Differential diagnosis of cPACNS.

**DIFFERENTIAL DIAGNOSIS OF PACNS**
**CNS-involvement in systemic inflammatory/rheumatic diseases** For example, Takayasu arteritis, polyarteritis nodosa, Kawasaki disease, Henoch-Schönlein purpura, Behçet disease, granulomatosis with polyangiitis, microscopic polyangiitis, systemic lupus erythematosus, juvenile dermatomyositis, inflammatory bowel disease, hemophagocytic lymphohistiocytosis, *ADA 2-*deficiency, *TREX1*-associated diseases (e.g., Aicardi-Goutieres syndrome)
**Secondary vasculitis to other causes** For example, in malignancy, infections, drugs
**Infectious encephalitis** For example, varicella zoster virus–post-varicella angiopathy
**Thromboembolic disease** For example, in coagulation disorders, hemoglobinopathies, coronary artery disease, congenital heart disease
**Reversible vasoconstriction syndrome**
**Moyamoya syndrome** Primary moyamoya MYMY1–MYMY6 (e.g., *RNF213*)
Secondary moyamoya syndrome, e.g., in Down syndrome, sickle cell anemia, neurofibromatosis type 1
**Neurometabolic diseases** For example, Fabry disease (*GLA*), homocystinuria
**Fibromuscular dysplasia**
**Migrainous infarction** (ICHD3: 1.4.3.)
**Genetic vasculopathies** For example, *NOTCH3, HTRA1*
**Genetic structural alterations of vessels** For example, *COL4A1, ACTA2, MOPD2*
**Structural vasculopathies** (causing mostly hemorrhagic stroke): For example, arterial dissection, arteriovenous malformations, aneurysm, cavernous malformation

### Laboratory Studies

As the clinical manifestations of cPACNS are rather diverse, precise diagnosis can be quite challenging because of the lack of vital laboratory tests or neuroimaging features. So far, no specific or sensitive screening parameters for cPACNS have been defined. Focused laboratory investigations are necessary to exclude secondary angiitis of CNS. The clinical findings such as newly acquired neurological deficit may guide further laboratory investigations such as serum inflammatory markers such as blood cell count, CRP, ESR, analysis of CSF, and neuroimaging (Table 3).

**Table 3:** Diagnostic assessments in cPACNS.

	**APP-cPACNS**	**APNP-cPACNS**	**AN-cPACNS**
Vessels	Internal carotid artery Circle of Willis	Internal carotid artery Circle of Willis	Small vessels
MRI	Arterial stenosis, changes in vessel caliber (TOF, MR angiography), vessel wall thickening, and contrast enhancement (contrast enhancement in T1 with fat saturation or black-blood impulse) Localization: often unilateral and bilateral focal or segmental often posterior cerebral artery involved	Arterial stenosis, changes in vessel caliber (TOF, MR angiography) vessel wall thickening. and contrast enhancement (contrast enhancement in T1 with fat saturation or black-blood impulse) Localization: often unilateral focal or segmental	Leptomeningeal enhancement (contrast enhancement in T1), ischemia (diffusion-weighted imaging, fluid-attenuated inversion recovery), impaired blood–brain barrier (contrast enhancement in T1)
Invasive angiography	Arterial stenosis, changes in vessel caliber	Arterial stenosis, changes in vessel caliber	No pathologic findings
Sonography	Arterial stenosis, changes in vessel caliber vessel wall thickening	Arterial stenosis, changes in vessel caliber vessel wall thickening	No pathologic findings
Laboratory test	In 50%, elevated CSF protein, CSF pleocytosis, elevated intracranial pressure; systemic inflammatory markers: sometimes mildly elevated	Systemic inflammatory markers typically unremarkable, CSF abnormalities possible but uncommon	In most patients, elevated CSF protein, CSF pleocytosis, increased intracranial pressure, in ~20% oligoclonal bands, sometimes increased systemic inflammatory markers
Biopsy	Not indicated/optional	Not indicated/optional	Indicated (preferred full-thickness biopsy targeting lesions): perivascular lymphocytic infiltration
Criteria for diagnosis	Clinical presentation, MRI, laboratory results	Clinical presentation, MRI, laboratory results	Brain biopsy and clinical presentation, MRI, laboratory results

Anemia, thrombocytosis, elevated liver enzymes, and low complement are frequent findings in PACNS. Cellucci et al. (16) showed that von Willebrand factor antigen (VWF) is a possible marker of disease activity in children with cerebral vasculitis. Raised inflammatory markers in serum and CSF are more commonly seen in AN-cPACNS. In children with large-vessel vasculitis, inflammatory markers in serum and CSF are often within the normal range in cases of non-progressive disease, whereas elevated inflammatory markers may be found in children with progressive course of vasculitis and in AN-cPACNS (15, 19, 22, 31, 38).

Analyses of CSF reveal further significant differences in pediatric PACNS compared to PACNS in adults. Typically signs of inflammation are found in approximately a third of the children with AN-cPACNS. The findings may be minor and non-specific, with only mild typically lymphocytic pleocytosis, elevated protein levels, or raised opening pressure. In adults with PACNS, CSF analyses disclose abnormal findings (lymphomonocytic pleocytosis, elevated protein levels, etc.) in the majority of the patients (32, 44).

Further investigations are needed to exclude other causes of vasculitis and to differentiate between primary and secondary CNS vasculitis in children. Underlying causes of secondary vasculitis are infections, rheumatic diseases, other systemic inflammatory conditions, and malignancies. The most common cause for secondary vasculitis of the CNS is infection. Infection due to varicella zoster virus (VZV) is the most common trigger for secondary CNS vasculitis in children (47). The diagnosis of post-varicella vasculitis is probable in cases with stroke occurring within 12 months of infection with VZV, positive VZV polymerase chain reaction, and VZV–immunoglobulin M serum-to-CSF ratio (48). Although rare, vasculitis of the CNS can follow infection with *M. tuberculosis*. Tuberculosis blood tests should be included in the laboratory diagnostic workup before starting immunosuppressive treatment (49, 50).

The diagnostic approach in children with newly diagnosed vasculitis of the CNS implies laboratory investigations to detect possible underlying autoimmune or autoinflammatory causes such as lupus erythematosus or systemic vasculitis. Screening for antibodies antinuclear antibodies, double-stranded DNA autoantibodies, and antineutrophil cytoplasmic antibodies should be performed. Especially in lupus, AIS is often due to underlying antiphospholipid syndrome. Therefore, screening for anticardiolipin autoantibodies and lupus anticoagulants is necessary. Depending on the patient history and clinical and laboratory findings, further investigations targeting underlying diseases such as juvenile dermatomyositis, juvenile scleroderma, Behçet disease, or inflammatory bowel diseases are needed. Besides these conditions for secondary central nervous vasculitis, vasculitis mimics should be considered. As a consequence of the rapid development in next-generation sequencing (NGS) technologies, several novel genes and phenotypes associated with vasculitis have been identified (Table 3). The increasing knowledge on molecular pathways has led to new insights into the pathophysiology of cerebral vasculitis and the design and development of new precise diagnostic tools. One example is the vasculitis due DADA2 or ADA2 deficiency (MIM#615688), where besides the key feature of CNS vasculitis, a variety of unspecific, systemic non-CNS symptoms such as polyarteritis and livedo reticularis are present (51, 52) This novel disease entity builds a further bridge to the autoinflammatory context in cerebral vasculitis. The increasing knowledge on genes and pathways in autoinflammatory diseases with involvement of the cerebral vessel as a key symptom will evolve classification and diagnostics of cerebral vasculitis over time.

### Coagulation Diagnostics

Thrombophilia diagnostics in case of cPACNS is not indicated (53). Neither inherited nor acquired thrombophilia such as the presence of antiphospholipid antibodies is associated with inflammatory CNS diseases (33, 54). The significance of genetic diagnostics by NGS in pediatric stroke is not clear yet (55). Concerning the hemostaseologic diagnostic workup at the manifestation of AIS the assessment of coagulation global tests [prothrombin time (Quick), activated partial thromboplastin time (aPTT), and fibrinogen- and d-dimer concentrations] is recommended to exclude a plasmatic coagulation disorder. Antithrombin should be measured and substituted if below the reference range, which is important especially for the anticoagulation with heparin. Moreover, the measurement of VWF may be a valuable biomarker because a decrease in VWF levels in serum seems to indicate improvement of cPACNS disease activity (16). Von Willebrand factor antigen levels increase in serum in response to endothelial injury or activation; therefore, monitoring of VWF levels may reflect disease activity in many vasculitis (56, 57). However, another study in pediatric PACNS found VWF levels to be a poor discriminator of disease activity, and a study of VWF in adults with antineutrophil cytoplasmic antibody–associated vasculitis observed the persistence of high levels of VWF when patients were considered to be in clinical remission (58, 59). Whether elevation of VWF solely represents endothelial activation or injury, or alternatively platelet activation and/or activation of the clotting cascade, is debatable (59).

### Brain Biopsy

As laboratory and neuroimaging findings are often non-specific in suspected AN-cPACNS, brain biopsy is the gold standard of diagnosis. The diagnostic value of brain biopsy is influenced by whether a lesional or non-lesional biopsy is performed and whether a full- thickness biopsy is obtained. Furthermore, parameters such as prolonged time to biopsy, previous corticosteroid treatment, and non–full-thickness samples were shown to reduce the diagnostic yield of brain biopsy in small vessel angiitis (27, 60). If inflammatory lesion is not accessible for biopsy, a non-lesional biopsy of the non-dominant temporal lobe is recommended. This procedure seems to equally succeed in yielding the diagnosis of AN-cPACNS in children (27, 31, 61), albeit the literature displays inconsistent results. Hajj-Ali et al. (37) reported a limited sensitivity and negative predictive value of histology in suspected PACNS. The presence of a radiological target was associated with a higher diagnostic yield (62).

The histologic findings in cPACNS differ from those in adults (24, 27, 61, 63). Tissue examination in pediatric small vessel angiitis reveals a lymphocytic vasculitis with intramural and perivascular inflammation and disruption of the vascular endothelium. The infiltrate of inflammatory cells is predominantly T-lymphocytic. In order to confirm the diagnosis of AN-cPACNS, a brain biopsy should be pursued, in particular as the diagnosis of AN-cPACNS leads to the initiation of a long-lasting potentially toxic immunosuppressive therapy. In selected cases, brain biopsy might be rejected from the parents because of the invasive nature of the procedure, the morbidity rate, or the uncertain yield in children. Limited experience with the disease and the interpretation of the results might be factors for restraints in pediatricians. Without a brain biopsy, the cerebral angiitis would be classified as probable PACNS (23), and the decision to initiate an immunosuppressive therapy is based on clinical, laboratory, and imaging data alone.

### Neuroimaging

Inflammation of blood vessels may affect abnormalities in both the vessel and the brain. Direct signs of cerebral vasculitis such as vessel wall thickening or vessel wall enhancement correspond to direct changes of the affected vessel. Indirect signs such as stenoses, including multiple stenosis and beading (alternating, short, regularly spaced segments of stenosis with short normal or dilated intervening segments); ischemic brain lesions; hemorrhage; and cerebral perfusion deficits represent changes secondary to vasculitis (40, 64). Imaging assessment is limited to medium and large vessels. Although small-vessel vasculitis may also cause indirect signs, it affects arteries beyond the spatial resolution of imaging techniques (64). Vessel wall inflammation leads to FCA regardless whether it is a primary vasculitis or secondary to a systemic cause or (septic) meningitis (20, 40). Unilateral inflammatory FCA has been recently classified as FCA–inflammation type, which includes TCA (40, 65).

A characteristic of childhood vasculitis is a distinct pattern of lesions compared to adults (40). It usually affects the proximal anterior circulation. In particular, the terminal internal carotid artery, media cerebral artery, and anterior cerebral artery are unilaterally affected.

Typically, multiple parenchymal lesions affect both gray and white matter. With the involvement of the lenticulostriate vessels, the lesions are often centrally located and involve the basal ganglia (40, 64, 66). The presence of parenchymal lesions is more important in children than in adults, who generally have a higher incidence of confounding foci of signal intensity abnormality. The imaging workup of a children vasculitis typically consists of MRI as the modality of choice. In some centers, CT is the initial imaging. Conventional angiography should be reserved for exceptional cases (40, 64, 67).

#### Magnetic Resonance Imaging

Magnetic resonance imaging is the most common imaging modality in the workup of cerebral vasculitis and the gold standard in the assessment of pediatric AIS and cerebral vasculitis. It combines a high tissue contrast with a differentiated visualization of the vessels and in particular the pathologically affected vessel wall. Repeated negative MRI is a strong negative predictor of cerebral vasculitis. Standard imaging should include T1- and T2-weighted sequences, as well as fluid-attenuated inversion recovery (FLAIR) sequences, diffusion-weighted imaging (DWI), susceptibility-weighted imaging, time-of-flight (TOF) MR angiography (MRA), and contrast material–enhanced T1-weighted imaging (66). T2 and FLAIR shows marked parenchymal lesions, and FLAIR improves lesion detection near the brain–CSF interface (66). The DWI detects acute infarction and helps to distinguish them from chronic changes. Contrast-enhanced T1-weighted images show not only parenchymal enhancement, but also leptomeningeal enhancement, which is even more evident in contrast-enhanced FLAIR (66, 68, 69). Susceptibility-weighted imaging supports the diagnosis of cerebral vasculitis by detecting associated microbleeds (70).

**Figure 2:**
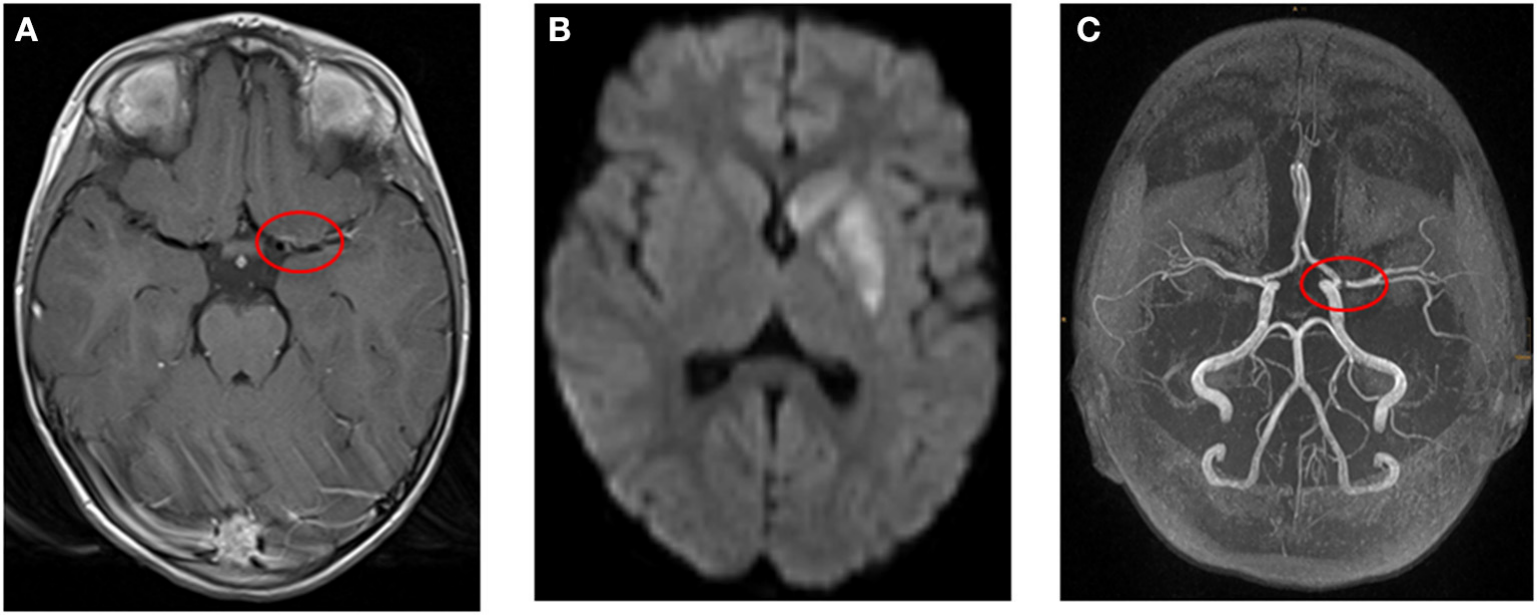
**(A–C)** Example for a 4-year-old girl with ischemic stroke due to large vessel vasculitis and right-sided hemiparesis and facial palsy. **(A)** Transversal T1-weighted MR image after contrast shows wall enhancement of the middle cerebral artery (red circle). **(B)** Transversal diffusion-weighted image (b1000) shows restricted diffusion in the left basal ganglia. **(C)** Time-of-flight MR angiography demonstrates stenosis of the middle cerebral artery (red circle, left).

**Figure 3:**
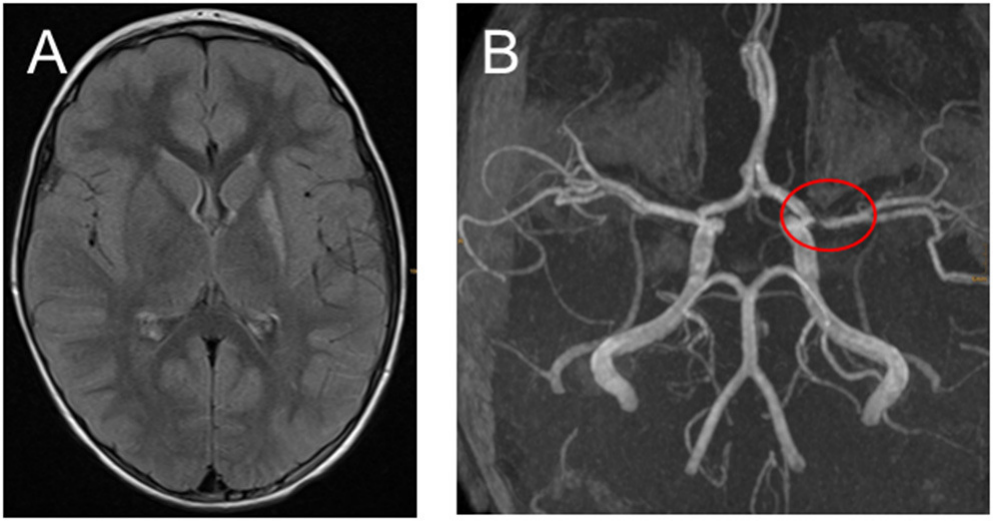
**(A,B)** Fifteen months after the ischemic stroke with only very mild hemiparesis remained and no stroke relapses. **(A)** Transversal fluid-attenuated inversion recovery images demonstrate high signal in the left-sided basal ganglia. **(B)** Time-of-flight MR angiography still showing middle cerebral artery stenosis.

Of particular importance is the visualization of the vessels. An inflammation of the vessels leads initially to a thickening of the vessel wall, which subsequently results to a stenosis of the lumen. This process may be associated with necrosis and later develop pseudoaneurysms (64). The TOF-MRA is best MRA for the visualization of intracranial vessels. It combines a high sensitivity for stenoses with a high spatial resolution, which can be further increased by 3-T MRI systems (64, 71, 72). Vessel wall imaging is a special MRI technique to visualize the pathologic changes of the vessel wall (73). It is a method to differentiate various causes of arteriopathy beyond conventional angiography (65, 67, 74). Black-blood T1-weighted imaging before and after gadolinium contrast may show vessel wall enhancement and indicate inflammation (73). Imaging without gadolinium contrast can be used to identify intramural hematoma and exclude FCA–dissection type as cause of AIS (65). As AN-cPACNS can show a multitude of different MRI findings such as leptomeningeal enhancement or ischemic lesions, the term “MRI negative” or “angiography negative” should be reconsidered in AN-cPACNS (Figures 2, 3).

#### Computed Tomography

Non–contrast-enhanced computed tomography (CT) is often the initial imaging in a child presenting with possible stroke (67). Computed tomography is less sensitive than MRI in the assessment of cerebral vasculitis (66). However, CT and CT angiography (CTA) can also be used to evaluate direct and indirect signs of vasculitis (66). Computed tomography angiography allows simultaneous visualization of the vessel wall and the lumen (75). It also demonstrates stenosis, occlusion, aneurysm, and concentric arterial wall thickening (75). Although CTA cannot depict small vessels, it may show vessel wall alterations, even before the lumen is affected in conventional catheter angiography (66). However, because of limited sensitivity for acute childhood AIS and concerns of radiation, CT is increasingly displaced by MRI in many centers as initial imaging (67, 76). Computed tomography with CTA of the head and neck may be preferred for children in critical clinical condition, with contraindication to MRI or in centers without MRI capabilities, or if sedation for MRI scan will delay diagnosis and subsequent therapy (67).

#### Conventional Angiography, Digital Subtraction Angiography

Digital subtraction angiography (DSA) shows changes that affect the lumen of the medium- to large-sized arteries. It has a better resolution than CTA and MRA and has advantages in the evaluation of smaller vessels (66, 77). The information provided by DSA is limited to the lumen and does not allow a direct evaluation of the vessel wall (64, 66, 77). Digital subtraction angiography is considered the gold standard; because of its invasive nature, radiation exposure, and minor morbidity, it is less frequently performed than MRA in many centers (40, 66). If MRA and CTA fail and strong suspicion of arteriopathy persists, DSA might help elucidate the stroke etiology (67).

## Therapy

The primary objective of chronic medication in cPACNS is secondary prevention of AIS relapses and/or neurocognitive impairment. As cPACNS is associated with a high morbidity and mortality, timely diagnosis and induction of immunosuppression are essential. Survival and neurological outcome depend on early diagnosis (15, 22) and prompt initiation of immunomodulatory therapy. The diagnosis of cerebral vasculitis is often a challenge for the treating pediatrician as cPACNS is a rare disease and exhibits marked disease overlap with inflammatory brain disease mimics. As for many rare diseases no evidence- or consensus-based therapy guidelines (or large clinical trials) are available. Particular attention should be given to the exclusion of cPACNS mimics (Table 2), especially systemic infections prior to immunosuppressive therapy. Concomitant symptomatic therapy in pediatric cerebral vasculitis focuses on the specific neurologic symptoms such as seizures, stroke, headache, psychiatric symptoms, and so on, and should be initiated immediately.

### Anticoagulation and Acute Recanalization Therapies

Treatment approaches concerning anticoagulation and platelet function inhibition in childhood PACNS vary significantly throughout the literature.

Current treatment recommendations for cPACNS and pediatric stroke are based on the guidelines of the American College of Chest Physicians and on some publications (78–82).

Before anticoagulation or platelet inhibition is commenced, the indication for lumbar puncture should be evaluated as antiplatelet and anticoagulant drugs increase the risk of hemorrhagic complication, for example, spinal hematoma (83).

In suspected cPACNS, the use of low-dose acetylsalicylic acid (ASA) is recommended as secondary prophylaxis. As an ASA “resistance” was described in children, but the clinical significance of this laboratory phenomenon has not been proven, a clear recommendation for platelet function testing to demonstrate a sufficient platelet function inhibition by ASA cannot be given (84, 85). In definite cPACNS, initial anticoagulation is started with low-molecular-weight heparin (LMWH) preferentially with enoxaparin or unfractionated heparin (UFH) at therapeutic doses. With enoxaparin, the target anti–Xa level should range from 0.5 to 1.0 anti-Xa units per milliliter measured 4–6 h after injection The UFH dose is adjusted according to the aPTT value with a target range of 60–80 s or an anti–Xa level ranging from 0.3 to 0.7. Because UFH and LMWH need sufficient levels of antithrombin, levels should be measured repeatedly together with aPTT or anti-Xa activities and substituted if below the reference range for adults (78).

Alternatively, in case of cPACNS with AIS, initially platelet function inhibition with ASA can be started (81). Acute treatment should last not < 1 week. For long-term treatment, we recommend the combination of ASA for ≥2 years with clopidogrel for 6–12 months (39). Despite increasing data that thrombectomy might also be beneficial for children suffering from thromboembolic AIS, there are no data supporting endovascular treatment in cerebral vasculitis. The risk of recanalization treatment for thromboembolic complications due to vasculitis has to be evaluated individually. According to the actual German guideline, for adults, thrombolysis and thrombectomy are not indicated in case of PACNS (33).

### Anti-inflammatory and Immunomodulatory Therapy

Currently, no evidence-based therapy guidelines for pediatric cPACNS are available. Current treatment concepts typically include glucocorticosteroids, cyclophosphamide, antiplatelet agents, and other immunosuppressive agents, differing in doses and treatment duration (38, 46, 86). As homogenous treatment protocols are still lacking, national study groups are consenting therapy recommendations.

The central aim of the systemic treatment strategy is a rapid and sustainable control of the inflammation by using potent anti-inflammatory approaches. Upon achievement of at least a partial remission, this induction therapy is generally followed by a maintenance therapy.

In patients with presumable non-progressive cPACNS, anti-inflammatory therapy is based on corticosteroids alone, as the vessel inflammation is considered to be of limited, monophasic course. These children usually receive initial pulse intravenous methylprednisolone, followed by oral prednisolone tapering over 3 months in addition to antithrombotic therapy (38, 86, 87). Because of the potential transient nature of non-progressive vasculitis, immunosuppressive therapy might be put on hold. A significant number of patients are not unequivocally to differentiate between progressive and non-progressive forms. In these cases or in cases of severe side effects of steroids, initiation of a therapy with mycophenolate mofetil (MMF) should be considered.

In cases of progressive disease, AN-cPACNS, or disease relapse, induction therapy is historically based on high-dose corticosteroids in combination with intravenous cyclophosphamide, in analogy to the treatment of systemic vasculitis of large vessels (39, 86, 88, 89). The most commonly used regimens are based on pulse intravenous methylprednisolone. Afterward, prednisolone should be initiated with steroid tapering over a period of at least 3–6 months. The duration of the tapering depends on the underlying subtype of the disease, the response to induction therapy, and side effects of steroids (86).

In addition to glucocorticosteroids, patients with progressive disease or small vessel PACNS were usually treated with intravenous pulses of cyclophosphamide every 4 weeks for 7 months in order to increase immunosuppression, thus leading to a higher rate of remission (38, 86). After this potent immunosuppressive induction therapy, maintenance treatment with low-dose corticosteroids, MMF or azathioprine is started. Maintenance therapy is typically given for 18 months (38, 39, 86). Concomitant anticoagulation therapy is mandatory. Given the potentially severe side effects of a cyclophosphamide therapy (e.g., hemorrhagic cystitis, myelosuppression, gonadal toxicity), alternative immunosuppressive strategies were tested. Despite the lack of randomized controlled trials and therapy guidelines, MMF seems to represent a potent and well-tolerated strategy for achieving and maintaining clinical remission in CNS vasculitis.

Salvarani et al. (46) demonstrated in a small case-series efficacy of MMF in adults with PACNS. Mycophenolate mofetil was shown to be effective and less toxic than other immunosuppressive strategies including cyclophosphamide. Further case reports illustrated similar results for MMF (87, 90). Based on these single case reports, five consecutive pediatric patients with pPACNS were treated with MMF after induction therapy with steroids at the Children's University hospital of Dresden. All patients showed a rapid and sustained clinical response to this efficacious and less toxic treatment (39).

Randomized prospective multicenter studies are warranted to finally answer the question of which treatment strategy may be the most efficacious and safest option.

## Conclusion

The heterogeneous nature and clinical course of cPACNS as a rare disease require an interdisciplinary diagnostic and treatment approach ideally in a tertiary center. International standardized diagnostic and therapy guidelines are needed as cPACNS contributes to a large portion of ischemic brain injury in children associated with high morbidity and mortality if misdiagnosed. Primary angiitis of the central nervous system in childhood differs in several parameters from primary cerebral angiitis in adulthood. Future efforts in clinical research should focus on the improvement of classification and algorithms for timely diagnosis of pediatric cerebral vasculopathy. Advancements in vascular imaging, for example, imaging of the arterial wall itself, may facilitate the differentiation of arteriopathies with distinct pathophysiologies. For the clinical perspective, we propose to restrict the classification of cPACNS to the affected vessel size [(1) medium and large-vessel vasculitis AP-cPACNS and (2) small-vessel vasculitis, AN-cPACNS] and exclude the time course for prompt initiation of immunomodulative and anticoagulative therapy. As for the clinician being confronted with the (sub-)acute symptoms and MRI pattern of a medium- or large-vessel vasculitis, the putative time course “progressive vs. non-progressive” remains unsolved at manifestation for most cases. In patients with typical presentation of a non-progressive course and without risk factors for a progressive course, the duration of therapy might be tailored and reduced swiftly. Chronic therapy may be adjusted to clinical symptoms and MRI patterns. Considerations might be given to stratify levels of certainty and include those in the classification of cPACNS. The rating may facilitate the complex balance of invasive diagnostic procedures and long-term putative toxic therapies in pediatric patients.

## Author Contributions

MS and MH equally contributed to all stages of this manuscript including conception and writing of the manuscript. NB, KE, GH, and RK wrote the review, performed the literature search, and reviewed subsequent drafts. MS and NB shared first authors as they have contributed equally. All authors contributed to the article and approved the submitted version.

## Conflict of Interest

The authors declare that the research was conducted in the absence of any commercial or financial relationships that could be construed as a potential conflict of interest.
